# A β-Lactam Antibiotic Dampens Excitotoxic Inflammatory CNS Damage in a Mouse Model of Multiple Sclerosis

**DOI:** 10.1371/journal.pone.0003149

**Published:** 2008-09-05

**Authors:** Nico Melzer, Sven G. Meuth, Delany Torres-Salazar, Stefan Bittner, Alla L. Zozulya, Christian Weidenfeller, Alexandra Kotsiari, Martin Stangel, Christoph Fahlke, Heinz Wiendl

**Affiliations:** 1 Department of Neurology, University of Würzburg, Würzburg, Germany; 2 Department of Neurophysiology, Medizinische Hochschule Hannover, Hannover, Germany; 3 Department of Neurology, Medizinische Hochschule Hannover, Hannover, Germany; 4 Department of Neurobiology, University of Pittsburgh School of Medicine, Pittsburgh, Pennsylvania, United States of America; University of Freiburg, Germany

## Abstract

In multiple sclerosis (MS) and its animal model experimental autoimmune encephalomyelitis (EAE), impairment of glial “Excitatory Amino Acid Transporters” (EAATs) together with an excess glutamate-release by invading immune cells causes excitotoxic damage of the central nervous system (CNS). In order to identify pathways to dampen excitotoxic inflammatory CNS damage, we assessed the effects of a β-lactam antibiotic, ceftriaxone, reported to enhance expression of glial EAAT2, in “Myelin Oligodendrocyte Glycoprotein” (MOG)-induced EAE. Ceftriaxone profoundly ameliorated the clinical course of murine MOG-induced EAE both under preventive and therapeutic regimens. However, ceftriaxone had impact neither on EAAT2 protein expression levels in several brain areas, nor on the radioactive glutamate uptake capacity in a mixed primary glial cell-culture and the glutamate-induced uptake currents in a mammalian cell line mediated by EAAT2. Moreover, the clinical effect of ceftriaxone was preserved in the presence of the EAAT2-specific transport inhibitor, dihydrokainate, while dihydrokainate alone caused an aggravated EAE course. This demonstrates the need for sufficient glial glutamate uptake upon an excitotoxic autoimmune inflammatory challenge of the CNS and a molecular target of ceftriaxone other than the glutamate transporter. Ceftriaxone treatment indirectly hampered T cell proliferation and proinflammatory INFγ and IL17 secretion through modulation of myelin-antigen presentation by antigen-presenting cells (APCs) e.g. dendritic cells (DCs) and reduced T cell migration into the CNS *in vivo*. Taken together, we demonstrate, that a β-lactam antibiotic attenuates disease course and severity in a model of autoimmune CNS inflammation. The mechanisms are reduction of T cell activation by modulation of cellular antigen-presentation and impairment of antigen-specific T cell migration into the CNS rather than or modulation of central glutamate homeostasis.

## Introduction

Multiple sclerosis (MS) is considered a paradigmatic autoimmune inflammatory disorder of the central nervous system (CNS) [1], [2]. Its animal model, experimental autoimmune encephalomyelitis (EAE), mimicks several aspects of the human disease [3]. Peripherally activated autoreactive T- and B-lymphocytes together with granulocytes and macrophages cross the blood-brain-barrier and migrate into the CNS parenchyma. This is followed by formation of inflammatory plaques in the CNS, that are mainly but not exclusively located in white matter [1]. The autoimmune attack against the myelin-sheath and oligodendrocytes (ODC) causes inflammatory demyelination accompanied by early neuronal cell death [4].

Within the inflamed CNS, proinflammatory cytokines such as TNFα und IFNγ are believed to cause an extracellular accumulation of glutamate: down-regulation of both, the predominant glial “Excitatory Amino Acid Transporter 2” (EAAT2) and glutamate metabolizing enzymes glutamine synthetase and glutamate dehydrogenase, impair the glial glutamate uptake capacity [5], [6]. Moreover, invading macrophages and T cells as well as resident microglia up-regulate glutaminase and secrete massive amounts of glutamate via different release-mechanisms [7], [8], [9]. Resulting excessive extracellular glutamate levels cause prolonged activation of calcium-permeable ionotropic glutamate receptors on neuronal and glial cells leading to excitotoxic CNS-tissue damage [10], [11]. Consistently, ionotropic glutamate receptor antagonists have proven to be effective in ameliorating the clinical course and CNS-tissue damage in EAE [10], [11], [12], thus making glutamate-mediated excitotoxicity an attractive target for future therapy of MS [13].

Rothstein et al. reported a pronounced long-term functional up-regulation of the glial glutamate transporter EAAT2 by β-lactam antibiotics thereby protecting neurons and glial cells from excitotoxicity in a variety of neurodegenerative disorders without interfering with regular synaptic transmission [14]. In order to develop strategies to dampen excitotoxic CNS damage in autoimmune CNS inflammation, we challenged the potential CNS-protective effect of ceftriaxone in MOG-induced murine EAE [15].

## Results

### A β-lactam antibiotic profoundly attenuates the clinical course of murine MOG-induced EAE

**Figure 1:**
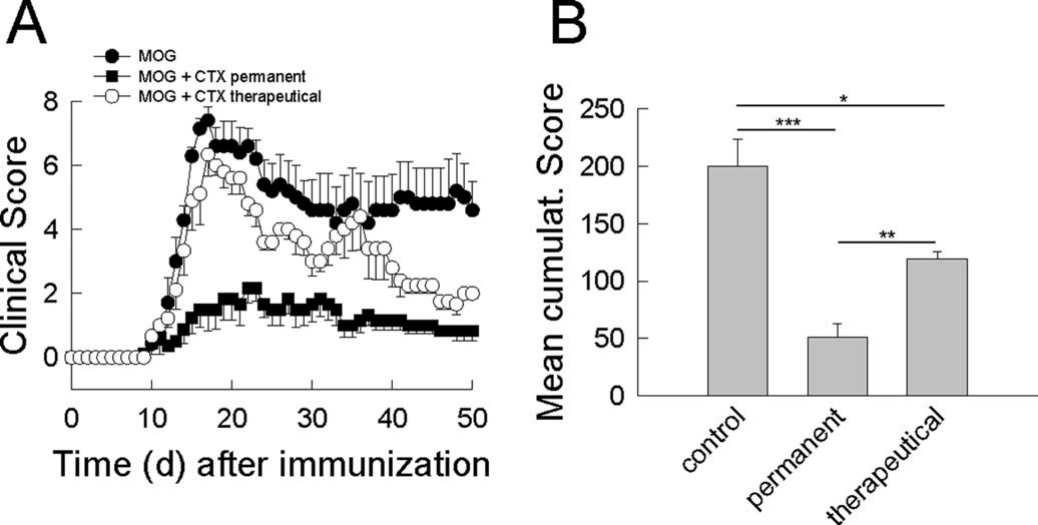
A β-lactam antibiotic profoundly attenuates the clinical course of MOG-induced EAE in mice. (A) Time course of neurological symptoms after immunization of WT C57BL/6 mice with a MOG peptide (MOG_35–55_). Mice were treated with ceftriaxone (200 mg/kg/d i.p.) either from the day of immunization (MOG+CTX permanent; filled squares) or from the individual onset of symptoms (MOG+CTX therapeutic; empty circles). MOG-immunized control mice (MOG) were injected with an equivalent volume of saline (MOG; filled circles). The degree of neurological impairment was assessed using a 10-point scoring system. (B) Mean cumulative score of MOG immunized mice treated with saline (control; n = 8) and with ceftriaxone from the day of immunization (permanent; n = 8) or from the individual onset of symptoms (therapeutical; n = 8). Differences between the 3 experimental groups are significant (control vs. permanent: p<0.001 ***; control vs. therapeutical: p = 0.05 *; permanent vs. therapeutical: p<0.01 **).

WT C57BL/6 mice were immunized with recombinant human MOG_35–55_ to induce EAE and injected daily with ceftriaxone (200 mg/kg/d i.p.) starting either from the day of immunization (MOG+ceftriaxone; n = 8; permanent treatment) or from the onset of neurological symptoms (MOG+ceftriaxone; n = 8; therapeutical treatment). MOG-immunized control mice (MOG; n = 8) injected daily with an equal volume of saline starting from the day of immunization developed a clinical course typical of EAE with an onset of neurological symptoms about day 10, a peak-clinical score of 7.4±0.4 at day 17 and a residual score of 4.6±0.9 (**Fig. 1A**, **Tab. 1**). Mice injected with ceftriaxone from the day of immunization showed a dramatically attenuated EAE course. Disease maxiumum was delayed, the peak score was significantly reduced (d 22: 2.2±0.5, p<0.001 ***) and the residual score significantly lowered (d 50: 0.8±0.3 (p<0.001 ***; **Fig. 1A**, **Tab. 1**). Mice treated with ceftriaxone from the onset of symptoms had a similar peak-clinical score of 6.3±0.7 (p = 0.23) 17 days post-immunization, but showed faster recovery of symptoms with a significantly lower residual score of 2.0±0.0 at day 50 (p = 0.04 *; **Fig. 1A**, **Tab. 1**). Non-immunized mice treated with ceftriaxone on a daily basis were used as an additional control. They neither developed any neurological symptoms nor any weight loss (**Tab. 1**). The mean cumulative clinical score was highly significantly different between the experimental groups (control vs. permanent: p<0.001 ***; control vs. therapeutical: p = 0.05 *; permanent vs. therapeutical: p<0.01 **; **Fig. 1B**, **Tab. 1**).

**Table 1:** Clinical parameters of the EAE course.

Number of C57BL/6 Mice	Immunization	Treatment (i.p.)	Day of Onset/Day of peak Score	Peak Score/Residual Score	Cumulative Score
8	200 µg MOG	saline	10/17	7.4±0.4/4.6±0.9	200±24
8	200 µg MOG	CTX (permanent)	10/22	2.2±0.5/0.8±0.3	51±12
8	200 µg MOG	CTX (therapeutical)	10/17	6.3±0.7/2.0±0.0	119±6
8	-	CTX	0/0	0.0±0.0/0.0±0.0	0±0
6	200 µg MOG	saline	10/17	7.0±0.7/3.8±0.3	153±18
6	200 µg MOG	saline+DHK	10/17	6.5±0.9/5.5±0.6	210±17
6	200 µg MOG	CTX	10/17	3.0±0.6/1.5±0.5	72±3
6	200 µg MOG	CTX+DHK	10/17	2.8±1.4/1.2±0.2	53±15
6	-	DHK	0/0	0.0±0.0/0.0±0.0	0±0

Disease incidence was 100% in all experimental groups of the experiments shown in Fig. 1 (white) and Fig. 5 (grey). CTX denotes ceftriaxone, DHK denotes dihydrokainate.

### Ceftriaxone does not influence total EAAT2 protein expression levels in the murine CNS

**Figure 2:**
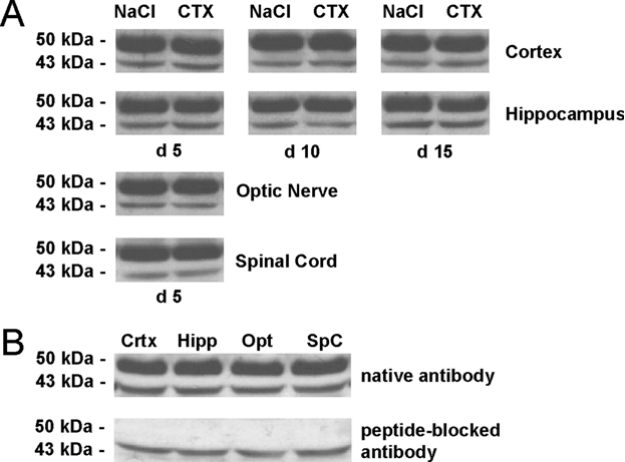
Ceftriaxone does not alter EAAT2 protein expression in several brain areas in mice. (A) Non-immunized mice were treated for 15 day either with ceftriaxone (CTX; 200 mg/kg/d i.p.) or an equal volume of saline (NaCl). No alteration of EAAT2 protein (50 kDa) expression levels could be observed in cortex, hippocampus, optic nerve and spinal cord after 5, 10 and 15 days of treatment assessed by western-blot analysis. (B) Specificity of antibody binding to the EAAT2 protein was confirmed using the immunogenic peptide to block the antibody (lower lane) that was used to detect the EAAT2 protein in the different brain areas after 5 day of treatment (upper lane). With the peptide-blocked antibody the 50 kDa band was virtually absent in samples from all brain areas tested, demonstrating specific binding of the antibody to the solubilized EAAT2 protein during western blotting. β-actin (43 kDa) was used as protein loading control.

We next tested whether upregulation of EAAT2 and subsequent reduction of inflammation-induced glutamate excitotoxicity was responsible for the beneficial effects of ceftriaxone in EAE. To assess the regulation of EAAT2 protein expression levels in the CNS in response to ceftriaxone treatment, non-immunized C57BL/6 mice were injected daily with ceftriaxone (200 mg/kg/d), sacrificed after 5, 10 or 15 days of treatment, and the EAAT2 protein content of several brain areas was assessed by western blot. During the experiment mice did not exhibit any neurological impairment (data not shown). Whole protein samples from the cortex, hippocampus, optic nerve and spinal cord were separated by SDS-page and EAAT2 protein was detected in an approximately 50 kDa band using an antibody specifically directed against the n-terminal amino acids 16–31 of the EAAT2 protein (**Fig. 2A, B**). Ceftriaxone-injection did not alter the protein expression level of EAAT2 in the brain areas examined.

### EAAT2-mediated glutamate uptake capacity in a rat primary mixed glial cell culture is not augmented by ceftriaxone

Unchanged EAAT2 protein expression as revealed by western blot analysis does not rule out a posttranslational effect of ceftriaxone on the number of functional transporters and the transport capacity of a single transporter protein in the plasma membrane.

**Figure 3:**
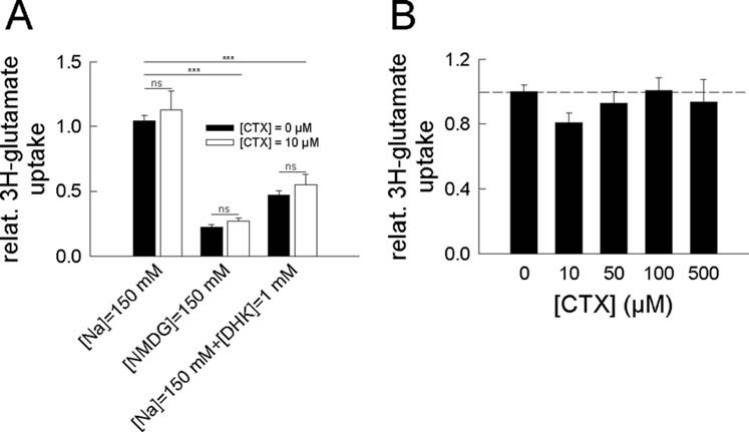
Dihydrokainate-sensitive radioactive glutamate uptake in a rat primary mixed glial cell culture is not influenced by ceftriaxone. (A) [3H]-glutamate (60 µM) uptake was measured in a rat primary mixed glial cell culture after 5 day of incubation with (white bars) or without (black bars) 10 µM ceftriaxone using either a NaCl-based external solution in the absence (left) or the presence (right) of 1 mM dihydrokainate or using a sodium-free NMDG-Cl-based external solution (middle). Substitution of external sodium by NMDG significantly reduced the uptake in the absence as well as in the presence of ceftriaxone (0.22±0.02 and 0.27±0.02, respectively; n = 3 trials consisting of 2 samples each; p<0.001 ***). Dihydrokainate lowered glutamate uptake to 0.47±0.03 in the absence and 0.55±0.08 in the presence of ceftriaxone (n = 3 trials consisting of 2 samples each; p<0.001 ***). (B) A ceftriaxone concentration-dependence of the [3H]-glutamate uptake could not be observed after 5 days of incubation with concentrations between 0 and 500 µM (p([ceftriaxone] = 0 µM vs. [ceftriaxone] = 500 µM) = 0.19; n = 3 trials consisting of 6 samples).

To assess the impact of ceftriaxone on the functional surface membrane expression level of EAAT2, we used radioactive glutamate uptake in a rat mixed primary glial cell culture. In a first set of experiments, glutamate uptake was determined after 5 days of incubation with 0 µM or 10 µM ceftriaxone using either a NaCl-based extracellular solution in the absence and the presence of 1 mM dihydrokainate or a sodium-free NMDG Cl-based extracellular solution to confirm the sodium-dependence of the transporter-mediated uptake (**Fig. 3A**). A ceftriaxone concentration of about 10 µM is usually reached in the cerebrospinal fluid of mice injected with 200 mg/kg/d i.p. and humans treated for infectious CNS disorders [16], [17]. Under all experimental conditions, 10 µM ceftriaxone caused no significant increase of glial glutamate uptake. However, with external NMDG, a profoundly reduced uptake could be observed in the absence as well as in the presence of 10 µM ceftriaxone (0.22±0.02 and 0.27±0.02, respectively; n = 3 trials consisting of 2 samples each; p<0.001 ***), demonstrating the sodium-dependence of the secondary-active glutamate uptake process. Moreover, with the EAAT2 specific inhibitor, dihydrokainate [18], present in the external NaCl-based solution, glutamate uptake was lowered to 0.47±0.03 in the absence and 0.55±0.08 in the presence of ceftriaxone (n = 3 experiments performed in duplicates; p<0.001 ***), indicating that glutamate uptake in the mixed glial cell culture is essentially mediated by EAAT2. Assuming the radioactivity measured after incubation in a sodium-free external solution to be due to unspecific background activity, the dihydrokainate-sensitive fraction of the total glutamate uptake is 0.80±0.05 in the absence and 0.85±0.1 in the presence of ceftriaxone, closely matching previous results [18], [19], [20], [21].

To rule out a considerable effect of higher ceftriaxone-concentrations, we measured glial glutamate uptake after incubation with ceftriaxone concentrations up to 500 µM (**Fig. 3B**). However, even at these concentration levels (very unlikely to be reached therapeutically in the CNS), we observed no significant alteration of radioactive uptake (p([ceftriaxone] = 0 µM vs. [ceftriaxone] = 500 µM) = 0.19).

### Electrical glutamate uptake currents are unaffected by ceftriaxone in transfected cells

**Figure 4:**
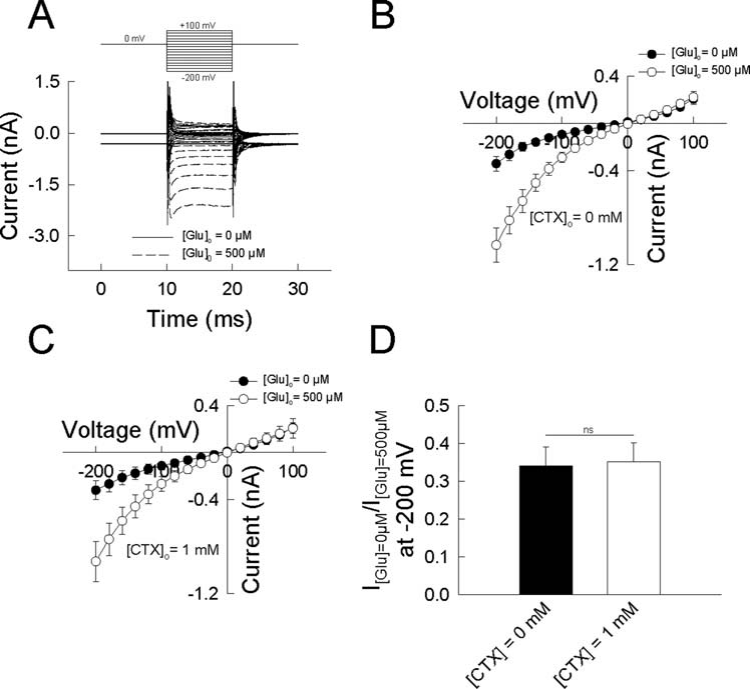
Ceftriaxone exerts no effect on the EAAT2-mediated electrical uptake current in a mammalian cell line. (A) Representative current traces recorded from a tsA 201 cell heterologously expressing hEAAT2 in the absence and the presence of 500 µM glutamate. The cell was held a 0 mV and 10 ms voltage-steps to potentials between −200 mV and 100 mV were applied. (B, C) Voltage-dependence of hEAAT2 mediated currents in the absence (filled circles) and the presence (empty circles) of 500 µM after pre-incubation with 0 mM (B) and 1 mM (C) ceftriaxone. (D) Inverse of the glutamate-induced current increase determined at −200 mV after pre-incubation with 0 mM (black bar) and 1 mM (white bar) ceftriaxone. Current ratios are not significantly different (p = 0.87; n = 6 individual cells, respectively).

In line with the lack of an effect of ceftriaxone on EAAT2-mediated glutamate uptake in our primary glia-cell culture experiments, we were unable to detect any increase of the electrical glutamate uptake current mediated by hEAAT2 expressed in a mammalian cell line. EAAT glutamate transporter mediate a secondary-active, stoichiometrically coupled and electrogenic transport process involving the inward translocation of three sodium ions and one proton and the outward translocation of one potassium ion per molecule of glutamate [19], [22], [23]. This results in a net inward transfer of two positive charges per transport cycle, that can be measured using whole-cell patch-clamp recording (**Fig. 4**).

We expressed hEAAT2 heterologously in tsA201 cells and performed whole cell patch-clamp recording using a KCl-based internal and a NaCl-based external solution in the absence and presence of 500 µM glutamate to allow for transporter cycling. Before experimentation, cells were incubated in ceftriaxone-free medium or in medium containing 1 mM ceftriaxone overnight for at least 12 h to assure detection of a rather long-term effect by covalent binding of ceftriaxone to the transporter protein. Moreover, ceftriaxone (1 mM) was also present in the recording solution of cells previously incubated with it to assure detection of a fast non-covalent binding effect of ceftriaxone.

Activation of the electrogenic glutamate transport by application of 500 µM external glutamate caused a current increase in the negative but not in the positive voltage range and a shift of the current reversal potential to more positive values (E_rev_-shift 14±4 mV at [ceftriaxone] = 0 mM; 10±3 mV, at [ceftriaxone] = 1 mM; p = 0.49; n = 6 cells, respectively) (**Fig. 4A, B, C**). The relative glutamate-induced current increase in the negative voltage range was indistinguishable in cells previously incubated for at least 12 h with 1 mM ceftiaxone and untreated cells (I([glu] = 0 µM)/I([glu] = 500 µM) at −200 mV = 0.34±0.05 without ceftriaxone (**Fig. 4B, D**); (I[glu] = 0 µM)/I([glu] = 500 µM) at −200 mV = 0.35±0.05 with 1 mM ceftriaxone (**Fig. 4C, D**); p = 0.87; n = 6 cells, respectively) (**Fig. 4D**).

### The clinical effect of ceftriaxone is preserved in the presence of the EAAT2-specific transport inhibitor dihydrokainate

**Figure 5:**
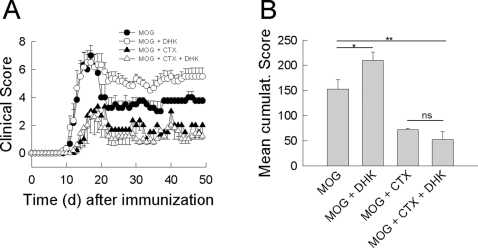
Ceftriaxone attenuates the clinical EAE course in mice in the presence of the EAAT2 transport inhibitor dihydrokainate. (A) Time course of neurological symptoms after immunization of WT C57BL/6 mice with human recombinant MOG. Mice were treated from the day of immunization with ceftriaxone alone (MOG+CTX; filled triangles; 200 mg/kg/d i.p.) or in combination with dihydrokainate (MOG+CTX+DHK; 10 mg/kg/d i.p.; empty triangles). MOG-immunized control mice were injected with an equivalent volume of saline alone (MOG; filled circles) or together with dihydrokainate (MOG+DHK; empty circles). The degree of neurological symptoms was assessed using a 10-point scoring system. (B) Mean cumulative score of mice from the different experimental groups. Differences between the experimental groups are significant (MOG vs. MOG+ceftriaxone: p = 0.004 **; MOG vs. MOG+ceftriaxone+dihydrokainate: p = 0.004 **; MOG vs. MOG+DHK: p = 0.05 *).

The lack of an effect of ceftriaxone on the *in vivo* EAAT2 protein expression level in mice as well as on the glial glutamate uptake capacity and the electrical uptake current *in vitro*, suggests a molecular target of ceftriaxone other than the glutamate transporter. To further test this assumption as well as to demonstrate the relevance of glutamatergic mechanisms in CNS inflammation we followed the course of EAE in the presence and absence of the EAAT2-specific transport inhibitor dihydrokainate (**Fig. 5**) [19]. In this setting, animals were also treated with ceftriaxone. MOG_35–55_-immunized mice were treated either with ceftriaxone (200 mg/kg/d) or an equal volume of saline from the day of immunization. In both groups a subset of mice (n = 6, respectively) received the EAAT2-specific transport inhibitor dihydrokainate at a concentration of 10 mg/kg/d i.p.

Treatment with dihydrokainate in MOG-immunized mice (MOG+DHK) alone neither changed the onset of symptoms nor the peak clinical score (6.5±0.9) at day 17 as compared to control animals (MOG; 7.0±0.7; p = 0.69). However, the residual score of the animals was significantly increased in dihydrokainate treated animals (5.5±0.6) compared to control mice (3.8±0.3; p = 0.05 *; **Fig. 5A**; **Tab. 1**). Of note, non-immunized mice treated with dihydrokainate (10 mg/kg/d) on a daily basis as control did not develop any neurological symptoms (data not shown). This clearly demonstrates the relevance of glutamatergic mechanisms for the permanent CNS damage and neurological disability in EAE.

In case ceftriaxon exerts its action via functional EAAT2 expression regulation, co-application of dihydrokainate will impede its clinical effect. MOG-immunized mice treated with ceftriaxone (MOG+ceftriaxone) again displayed a significantly attenuated clinical EAE course with a reduced peak score of 3.0±0.6 (p<0.01 **) 17 days after immunization and a residual score of 1.5±0.5 (p = 0.01 *; **Fig. 5A**; **Tab. 1**). However, mice co-injected with dihydrokainate (MOG+ceftriaxone+DHK) exhibited an indistinguishable clinical course with a peak score of 2.8±1.4 (p = 0.80) and a residual score of 1.2±0.2 (p = 0.51; **Fig. 5A**; **Tab. 1**).

The mean cumulative clinical score showed similar differences (**Fig. 5B**). Treatment with dihydrocainate alone significantly exacerbated the score but did not alter the beneficial effects exerted by ceftriaxone treatment (MOG vs. MOG+ceftriaxone: p = 0.004 **; MOG vs. MOG+ceftriaxone+dihydrokainate: p = 0.004 ** ; MOG vs. MOG+DHK: p = 0.05 *; **Fig. 5B**, **Tab. 1**).

### Immunological effects of β-lactam treatment: ceftriaxone reduces CD4^+^ T cell migration into the CNS

Ceftriaxone treatment delayed disease onset and ameliorated disease severity in EAE animals. We asked whether this β-lactam antibiotic might influence lymphocyte trafficking and entry of T cells into the CNS, an effect that could at least partially explain the observed findings and has been described for tetracyclines [24].

**Figure 6:**
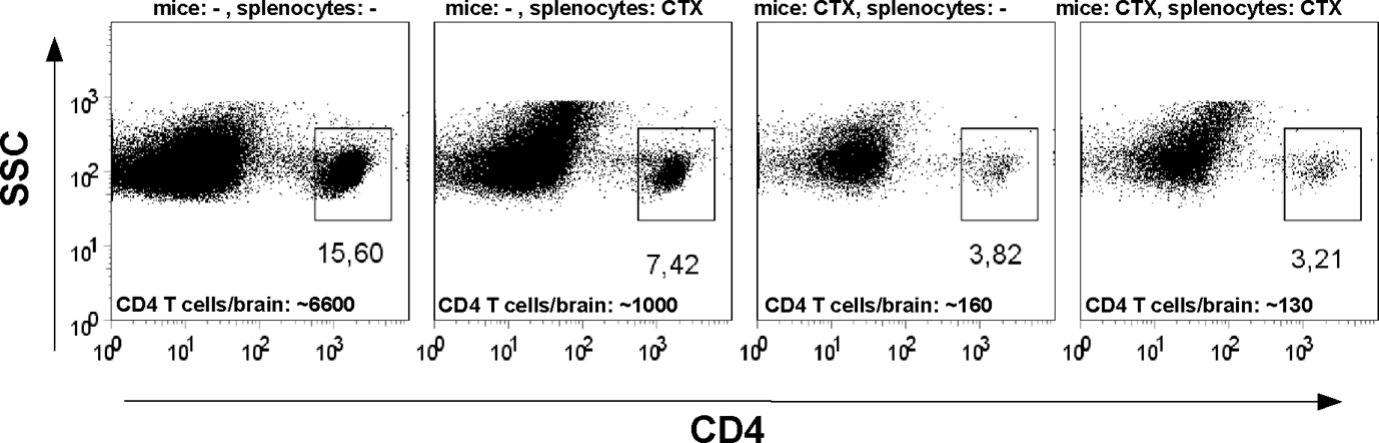
CNS invasion of neuroantigen-specific T cells is impaired by ceftriaxone. Splenocytes from TCR-transgenic 2D2 mice were stimulated for 5 days with MOG peptide (20 µg/ml) in the presence or absence of 500 µM ceftriaxone *in vitro* and adoptively transferred into WT C57BL/6 mice (3×10^6^ splenocytes/mice) pre-treated for 5 days with or without ceftriaxone (200 mg/kg i.p.). Dot plot show numbers of CNS invasive CD4^+^ T cells analysed 4 days after transfer using whole-brain FACS analysis. Mean absolute numbers of T cells/brain calculated from 3 to 4 mice pooled per experimental group are indicated in each histogram.

To directly test the impact of ceftriaxone on T cell penetration into the CNS *in vivo*, we performed an adoptive transfer of activated MOG-reactive CD4^+^ T cells obtained from TCR-transgenic 2D2 mice [25]. After incubation of 2D2 splenocytes with MOG (20 µg/ml) in the presence or absence of 500 µM ceftriaxone *in vitro*, splenocytes were adoptively transferred into WT C57BL/6 recipient mice (3×10^6^ splenocytes/mice) pre-treated for 5 days with or without ceftriaxone (200 mg/kg i.p.). 4 days post-transfer, numbers of CNS-invasive T cells were analysed using whole-brain FACS analysis (**Fig. 6**). Adoptive transfer of untreated splenocytes into untreated mice resulted in a roughly 40 to 50fold increase in the number of CD4^+^ T cells (about 6600/brain) compared to naïve WT mice (about 150/brain, data not shown). Surprisingly, presence of ceftriaxone during *in vitro* activation of T cells caused a 6 to 7fold reduction in the number of T cell in the CNS of untreated mice (about 1000/brain). Pre-treatment of mice with ceftriaxone before transfer of untreated T cells reduced CD4^+^ T cell numbers in the CNS to levels of naïve animals as did both, treatment of T cells and pre-treatment of mice together (about 150/bain). These findings indicate a considerable lasting effect of ceftriaxone on the T cell invasion into the CNS. However, we cannot completely rule out an effect of ceftriaxone on peripheral T cell re-stimulation after transfer due to pre-treatment of mice *in vivo* similar to that observed upon *in vitro* activation of T cells in the presence of ceftriaxone (**Fig. 6**).

### Ceftriaxone impairs T cell activation and antigen-specific cytokine production via modulation of antigen-presentation by APCs

Next, we asked, whether ceftriaxone exerts direct effects on immune cells thus explaining the beneficial effects in preventing EAE, ameliorating recovery and reducing the number of CNS invasive T cells *in vivo*.

**Figure 7:**
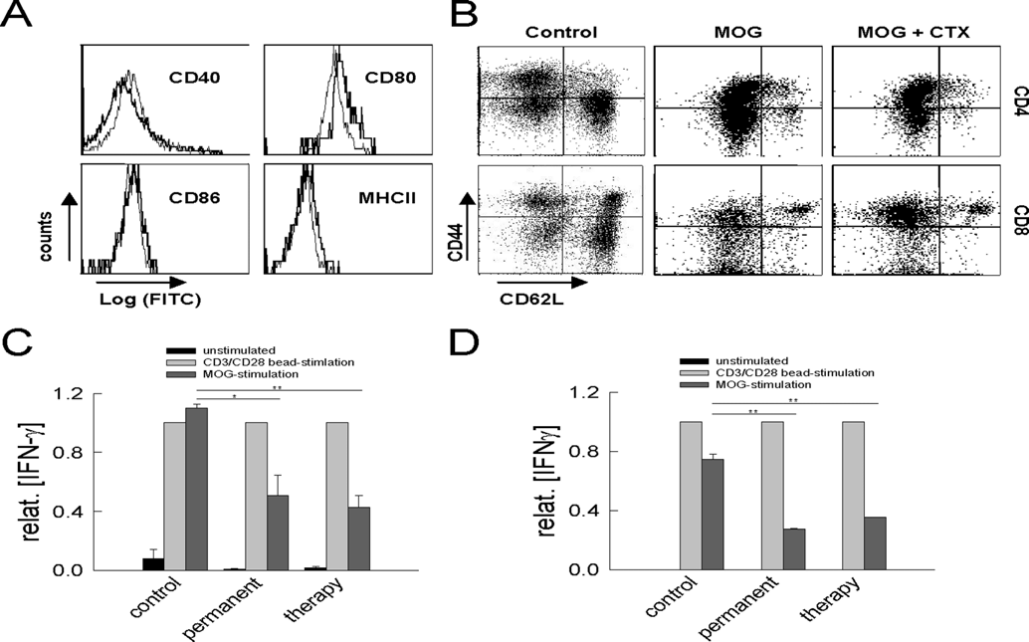
Ceftriaxone does not modulate phenotypical but functional properties of peripheral immune cells. (A) FACS-analysis of the activation markers CD40 (upper left), CD80 (upper right), CD86 (lower left) and MHCII (lower right) on CD11b^+^ CD11c^+^ APCs from spleen of untreated (thick lines) and ceftriaxone-treated (thin lines) MOG-immunized mice at the disease maximum. Geometric mean fluorescence intensities of all marker were similar between experimental groups. (B) Immunophenotyping of CD4^+^ (upper panels) and CD8^+^ (lower panels) T cells from spleen of non-immunized (left panels) as well as untreated (middle) and ceftriaxone-treated (right) MOG-immunized mice. Relative fractions of T cells as assed by CD40 and CD62L expression were similar between experimental groups. (C, D) MOG-recall experiments performed with splenocytes from untreated as well as permanently and therapeutically treated MOG-immunized mice at the disease maximum (C) and in the residual state (D) in the total absence of ceftriaxone *in vitro*. MOG-specific supernatant IFNγ-levels were significantly reduced relative to antigen-independent CD3/CD28 bead-stimulation in samples from MOG-immunized mice treated with ceftriaxone as compared to untreated MOG-immunized mice at the disease maximum (p (permanent) = 0.02 *; p (therapeutical)<0.01 **) and the residual state (p (permanent)<0.01 **; p (therapeutical)<0.01 **; n = 3 samples out of 3 animals, respectively). There was no difference whether mice were treated permanently or only after disease onset.

First, we performed immunophenotyping of peripheral CD11b^+^ CD11c^+^ antigen-presenting cells (APCs; **Fig. 7A**) and CD4^+^ and CD8^+^ T cells (**Fig. 7B**) derived from spleens of MOG-immunized mice treated permanently with ceftriaxone (200 mg/kg/d) or saline at the disease maximum (day 17). APCs from both groups displayed similar expression of various markers of maturation and antigen-presentation (CD40, CD80, CD86 and MHC II; **Fig. 7A**). Furthermore, distribution of T cell subsets (CD4: 18% vs. 13%; CD8: 11% vs. 12%; **Fig. 7B**) and their immunophenotype in terms of CD44 and CD62L cell surface expression (**Fig. 7B**) were similar between untreated and ceftriaxone-treated animals.

However, both ceftriaxone treatment-groups showed a significantly reduced production of the proinflammatory cytokine IFN-γ in response to MOG-peptide in specific recall experiments at the disease maximum (**Fig. 7C**) and the residual state (**Fig. 7D**). In this set of experiments, splenocytes from treated and untreated MOG-immunized mice were isolated, re-incubated with MOG *in vitro* in the absence of ceftriaxone and supernatant IFNγ-levels were assessed (**Fig. 7C, D**). MOG-specific IFNγ-levels were significantly reduced relative to antigen-independent CD3/CD28 bead-stimulation in samples from MOG-immunized mice treated with ceftriaxone as compared to untreated MOG-immunized mice at the disease maximum (p (permanent) = 0.02 *; p (therapeutical)<0.01 **) and the residual state (p (permanent)<0.01 **; p (therapeutical)<0.01 **; n = 3 samples out of 3 animals, respectively). There was no difference whether mice were treated permanently or only after disease onset (**Fig. 7C, D**).

**Figure 8:**
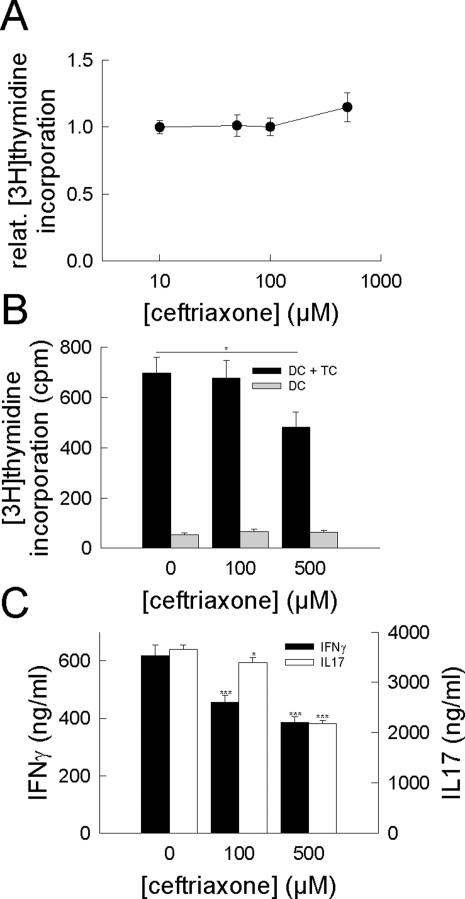
Reduced T cell response is due to ceftriaxone-induced modulation of cellular antigen-presentation. (A) Ceftriaxone concentration-dependence of CD3/CD28 stimulation induced proliferation of murine CD4^+^ T cells. Ceftriaxone does not inhibit [3H]thymidine incorporation in T cells (p([ceftriaxone] = 0 µM vs. [ceftriaxone] = 500 µM) = 0.12; n = 6 respectively). (B) Proliferation of murine CD4^+^ T cells (TCs) cocultured with dendritic cells (DCs) previously loaded with MOG peptide (50 µg/ml) in the absence and presence of different ceftriaxone concentrations. MOG-preincubation of dendritic cells in the presence of ceftriaxone impaired subsequent proliferation of T cells (p([ceftriaxone] = 0 µM vs. [ceftriaxone] = 500 µM): p = 0.05 *; n = 6). (C) Ceftriaxone concentration dependence of supernatant IFNγ and IL17 levels from the experiment described in (B). MOG-preincubation of dendritic cells in the presence of ceftriaxone lowered IFNγ and IL17 levels in a concentration dependent manner (p([ceftriaxone] = 0 µM vs. [ceftriaxone] = 500 µM): IFNγ: p<0.001 ***, IL17: p<0.001 ***; n = 6 respectively).

MOG-antigen-specific cytokine-secretion depends both on the efficacy of antigen-presenting cells (APCs) as well as on the activation of T cells. To dissect whether the observed effects by ceftriaxone are operative at the levels of modulated antigen-presentation or directly targets T cells we firstly examined the effect of ceftriaxone on T cell proliferation independent from APCs. CD4^+^ T cells were isolated from untreated, non-immunized mice and stimulated *in vitro* using CD3/CD28 bead-stimulation in the absence and presence of various ceftriaxone concentrations (up to 500 µM; **Fig. 8A**). Ceftriaxone concentrations used resemble those found in human and rodent blood serum after intravenous application [16], [17]. Stimulated cell proliferation assessed by radioactive thymidine uptake of murine T cells was not influenced by ceftriaxone (p([ceftriaxone] = 0 µM vs. [ceftriaxone] = 500 µM): murine p = 0.12; human p = 0.70; n = 6 respectively; **Fig. 8A**).

To investigate the potential influence of ceftriaxone on antigen-presentation, we incubated cultured dendritic cells (DCs) with MOG peptide (50 µg/ml) in the absence and presence of various ceftriaxone concentrations for 12 h, washed and re-incubated them for 3 days with CD4^+^ T cells from MOG-immunized mice at the disease maximum. Afterwards, T cell proliferation (**Fig. 8B**) and supernatant levels of IFNγ, IL17 (**Fig. 8C**), IL2 and IL4 were determined. MOG-antigen presentation by DCs was altered in the presence of ceftriaxone and subsequently hampered T cell proliferation (p([ceftriaxone] = 0 µM vs. [ceftriaxone] = 500 µM): p = 0.05 *; n = 6) and antigen-specific IFNγ and IL17 cytokine secretion (p([ceftriaxone] = 0 µM vs. [ceftriaxone] = 500 µM): IFNγ: p<0.001 ***, IL17: p<0.001 ***; n = 6 respectively) in a dose-dependent manner. Levels of IL2 and IL4, however, remained unaltered (data not shown). These experiments strongly suggest that ceftriaxone dampens autoimmune CNS inflammation by altering antigen-presentation and related activation of myelin-specific T cells.

## Discussion

Glutamate excitotoxicity is a dominant feature contributing to lesion pathogenesis and neuronal degeneration in multiple sclerosis [10], [11], [12]. In 2005 Rothstein et al. proposed that β-lactam antibiotics such as ceftriaxone exert a profound neuroprotective effect in pathological CNS conditions involving glutamate excitotoxicity by functionally up-regulating glial glutamate transporter expression [14]. We here challenged this mechanism by using ceftriaxone i.p. for the treatment of EAE, a mouse model of multiple sclerosis. In this model the pathological role of excitotoxicity has been confirmed through the amelioration of the clinical course by ionotropic glutamate receptor blockers [10], [11], [12]. We show that ceftriaxone exerted a profound attenuation of the clinical EAE course when applied in a preventive manner from the day of immunization. Furthermore, ceftriaxone also exerted beneficial effects when applied therapeutically after the onset of neurological symptoms. Mechanistic and functional experiments demonstrated that the beneficial effect of ceftriaxone was preserved *in vivo* in the presence of dihydrokainate, a EAAT2 specific transport inhibitor, indicating a key target mechanism of cetriaxone other than glutamate transporter expression regulation. Dihydrokainate alone caused a substantial aggravation of the clinical EAE course thereby demonstrating the need for sufficient glial glutamate uptake during an autoimmune inflammatory challenge and indirectly confirming the role of glutamate excitotoxicity in EAE pathogenesis. However, ceftriaxone did neither affect total EAAT2 protein expression levels in several brain regions *in vivo*, nor dihydrokainate-sensitive glutamate uptake in a primary glia cell culture and electrical uptake currents mediated by hEAAT2 in a heterologous expression system.

Moreover, ceftriaxone had no direct effect on T cell proliferation and their transendothelial migration in a blood-brain-barrier model. However, it indirectly hampered their proliferation and proinflammatory INFγ and IL17 secretion through modulation of myelin-antigen presentation by antigen-presenting cells. These observations explain the tremendous effects of ceftriaxone in modulating disease course and accelerating recovery in a model of autoimmune CNS inflammation.

Rothstein et al. reported the long-lasting, functional up-regulation of both EAAT2a and its c-terminal splice variant GLT1b [14], [26] in astrocytes from rats treated with ceftriaxone through binding to the EAAT2 promoter even after 3 months of treatment. In a recent study Lee et al. reported an augmented transcription in human astrocytes through NF-κB-mediated activation of the EAAT2 promoter by ceftriaxone [27], whereas Tian et al. reported EAAT2 translation to be increased by penicillin and ampicillin [28]. Ceftriaxone prevented neuronal cell death upon oxygen-glucose deprivation, an *in vitro* model of cerebral ischemia [14]. Furthermore, motoneuron loss induced by glutamate transporter inhibitors in spinal cord slice cultures was diminished by drug treatment and ceftriaxone caused motoneuron preservation associated with prolonged survival in G93A SOD1 mice [14]. In this mouse model of ALS glutamatergic excitotoxicity through altered expression of glutamate transporters and protection by glutamate receptor blockade has been demonstrated, similar to studies in the EAE model for multiple sclerosis [29], [30], [31], [32]. Moreover, regulation of EAAT2 expression and function by ceftriaxone has also been described by others in models of cerebral ischemia [33], [34], [35] and Huntington's disease [36].

Our study clearly demonstrated, that ceftriaxone exerted significant effects in attenuating autoimmune CNS inflammation and confirmed the relevance of glutamate transporter systems for permanent neural damage and neurological disability. Ceftriaxone treatment however did not affect transporter translation, post-translational trafficking to and from the plasma membrane and transporter function within the membrane: we were unable to detect any alteration of EAAT2 expression after 5, 10 and 15 days of treatment refuting a time dependence of expression regulation seen in the study by Chu et al. [33]. As EAAT2 expression continuously increases during developmental CNS maturation and is low in the early postnatal period [37], the missing effect of ceftriaxone on glial uptake in the mixed glial cell culture obtained from newborn rats may reflect reduced sensitivity of the EAAT2 promotor to β-lactam antibiotics. However, the vast majority of glial uptake was dihydrokainate sensitive and therefore predominantly mediated by EAAT2. Furthermore, adult mice were used in the EAE experiment and for the regional expression study, arguing against a developmental restriction of EAAT2 expression regulation in our study. Lipski et al [34] suggested a direct influence of ceftriaxone on the EAAT2 glutamate transport rate. Hence, we closer examined the effect of cetriaxone on the hEAAT2 electrical uptake current in a mammalian cell line, as all β-lactams potentially modify the conformation of membrane proteins by covalently binding to lysine or histidine residues exposed to the outer surface after spontaneous hydrolysis of the lactam ring [38], [39]. However, glutamate-evoked, hEAAT2-mediated inward currents, largely representing electrogenic glutamate transport, were not changed after over-night incubation with 1 mM ceftriaxone, refuting the idea of a slow covalent binding of ceftriaxone to the transporter. As 1 mM ceftriaxone was also present in the extracellular recording solution, a fast non-covalent binding effect on the transporter function also seems unlikely.

In search for other explanations than glutamate transporter expression regulation, we considered a β-lactam effect on trafficking of lymphocytes across the blood-brain-barrier, as demonstrated for tetracyclines [24]. We observed a dramatic reduction in the number of activated MOG-reactive CD4^+^ T cells that entered the CNS after adoptive transfer into mice pretreated with ceftriaxone to levels of naïve mice. This points towards a substantially impaired T cell trafficking into the CNS through the β-lactam. However, antigen-stimulation of MOG-reactive CD4^+^ T cells in the presence of ceftriaxone also considerably lowered the number of T cells detected in the CNS after transfer into untreated mice. This argues for a β-lactam effect on T cell activation and/or antigen presentation, that might also contribute to the dramatic effect of adoptively transferring untreated T cells into pretreated mice. However a ceftriaxone effect on T cell activation and/or antigen presentation due to systemic pre-treatment is expected to be modest compared to the *in vitro* effect of ceftriaxone on T cell stimulation in view of the short half-life time [17] and the fact that ceftriaxone treatment of mice was terminated before transfer of splenocytes.

We investigated the effects of ceftriaxone on immune cells. We found that ceftriaxone treatment in the EAE-model, caused a suppression of the peripheral T cell response in addition to its impact on lymphocyte trafficking. While distribution of peripheral T cell subsets and CD11b CD11c APCs was unchanged, ceftriaxone-treated mice displayed significantly lower MOG-specific IFNγ production upon antigen recall. Ceftriaxone altered antigen-presentation by professional APCs, as assessed by antigen-recall experiments measuring MOG-specific T cell responses after stimulation with syngenic DCs pretreated with ceftriaxone. Incubation of DCs with ceftriaxone lowered antigen-specific proliferation and secretion of proinflammatory cytokines IFNγ and IL17. This effect was clearly dose-dependent and occurred at concentrations found in humans treated with ceftriaxone intravenously under clinical conditions [16], [17]. After spontaneous opening of the lactam ring β-lactam antibiotics are able to covalently bind to soluble or membrane-bound proteins or peptides such as the MOG-peptide used for immunization [39] leading to processing and MHC-restricted presentation of β-lactam modified peptides (processing-dependent pathway) [39]. Alternatively, ceftriaxone might bind directly to the immunogenic peptide within MHC molecules (processing-independent pathway) [39]. In both cases, MHC-restricted presentation of chemically modified peptides by APCs might profoundly alter the subsequent T cell response in a dose-dependent manner [40]. Moreover, β-lactams have been reported to directly bind to IFNγ and other cytokines and inhibit their activity [41], [42], [43], an effect that might contribute to attenuation of EAE by ceftriaxone *in vivo*. However, all *in vitro* cytokine-assays have been performed from ceftriaxone-free supernatants, excluding artificially altered cytokine levels obtained from these experiments.

Clinical severity of MOG-induced EAE directly correlates with the number and degree of activation of proinflammatory, encephalitogenic T cells invading the CNS [15], [44]. To enter the CNS parenchyma naïve T cells need to be primed in secondary lymphatic organs and reencounter their cognate antigen presented by DCs located in the perivascular Virchow-Robin spaces [45], [46]. Hence, pathways or agents impeding T-cell activation and proliferation in the pre-clinical phase delay disease onset or lower severity of clinical symptoms, as observed in animals treated permanently with ceftriaxone [13]. However, ceftriaxone was also effective when applied in a therapeutic setting: animals treated after the onset of disease show significantly improved recovery of symptoms when treated with ceftriaxone. Ceftriaxone has a very good penetration over the blood-brain-barrier, reaching optimal concentrations in the CNS under therapeutic and non-toxic conditions. It is thus safe to speculate that drug-induced alteration of antigen-presentation and related reduction of T cell reactivation is operative also in the CNS parenchyma under conditions of ongoing autoimmune CNS inflammation where antigen-presentation is largely provided by microglia [45], [47], [48].

In summary, we demonstrate that a β-lactam antibiotic attenuates autoimmune encephalomyelitis, a model of multiple sclerosis. Ceftriaxone obviously shows no significant modulation of central glutamate homeostasis under the given experimental conditions. However, ceftriaxone impairs invasion of myelin-antigen specific T cells into the CNS parenchyma and reduces their activation and cytokine production through modulation of antigen-presentation by APCs. Antigen-presentation and T cell (re-)stimulation by distinct APC populations is required repeatedly during the initiation and perpetuation of autoimmune neuroinflammation, thus explaining the beneficial effects of ceftriaxone in EAE under preventive and therapeutic treatment conditions. Our findings contribute to the understanding of the mechanism of action of β-lactam antibiotics and have implications for considering these agents in attenuating T cell-mediated autoimmune disorders.

## Materials and Methods

### Induction, evaluation and treatment of EAE in C57BL/6 mice

Female WT C57BL/6 mice (Charles River Laboratories, Sulzfeld, Germany) were kept under pathogen-free conditions and had access to food and water ad libidum. All experiments were conducted according to the German law of animal protection and were approved by local authorities.

To induce EAE C57BL/6 mice between 6 to 8 weeks of age were actively immunized using a peptide consisting of amino acids 35–55 of myelin oligodendrocyte glycoprotein (MOG_35–55_, EVGWYRSPFSRVVHLYRNGK; synthesized and HPLC purified by R. Volkmer, Charité, Berlin, Germany). Mice were injected subcutaneously with 200 µg of MOG_35–55_ peptide emulsified with complete Freund's adjuvant (CFA; Sigma-Aldrich, München, Germany) containing Mycobacterium tuberculosis (5 mg/ml; Difco, Detroit, MI, USA). Control animals were immunized with an equivalent volume of CFA emulsion that did not contain MOG_35–55_ peptide. Additionally, all mice were injected intraperitoneally with pertussis toxin (400 ng; List Biological Laboratories, Campell, CA, USA) at the time of immunization and 2 days later.

Mice were evaluated on a daily basis for changes in body weight, overt signs of illness, and clinical signs of EAE by two blinded investigators using the following 10-point scoring system (**Fig. 1**, **5**): 0, no neurological signs; 1, distal tail limpness; 2, full tail limpness; 3, beginning broad-based gait with mild ataxia; 4, severely broad-based gait and pronounced ataxia; 5, mild hind limb paraparesis; 6, severe hind limb paraparesis; 7, hind limb paraplegia; 8, tetraparesis; 9, tetraplegia, moribund; and 10, death attributable to EAE. Mean cumulative clinical scores were determined as the mean of the cumulative scores of all mice of a certain experimental group (**Fig. 1B**, **5B**, **Tab. 1**). Days of disease onset and peak clinical score were determined from the time course of mean clinical scores of a certain experimental group (**Tab. 1**).

Mice were treated in various combinations with ceftriaxone (200 mg/kg/d i.p.; Fresenius, Bad Homburg, Germany) and/or dihydrokainate (10 mg/kg/d i.p.; Sigma-Aldrich, München, Germany) both dissolved in water as indicated in results.

### Western blotting of whole mouse brain protein

Non-immunized WT C57BL/6 mice were treated with ceftriaxone (200 mg/kg/d i.p.) or an equal volume of saline, sacrificed after 5, 10 or 15 days of treatment and lysates from cortex, hippocampus, optic nerve and spinal cord were used for western blotting procedures. Brain tissue was washed with ice-cold PBS, resuspended in lysis buffer (PBS containing 1% Triton X-100 and protease inhibitor cocktail (Roche Diagnostics, Germany)) and solubilized by sonification on ice. Cell lysates were centrifuged and protein content in the clarified supernatant was measured using the Bradford reaction. Samples containing equal amounts of protein were subjected to 10% SDS-PAGE, followed by transfer to nitrocellulose membranes. Protein transfer was visualized by Ponceau S staining and membranes were then blocked with PBS containing 0.05% Tween 20 and 5% dry milk. The membranes were probed with rabbit anti-EAAT2 (1∶50; Merck Chemicals, Darmstadt, Germany) directed against n-terminal amino acids 16–31 of the transporter to assure detection of both EAAT2a and –b and rabbit anti β-actin (1∶200; Santa Cruz Biotechnology, Heidelberg, Germany) as protein loading control. The secondary antibody was HRP-conjugated donkey anti-rabbit (1∶3000; Amersham, Buckinghamshire, UK). The antibody reaction was detected by enhanced chemiluminescence reaction (ECL, Amersham, Buckinghamshire, UK) (**Fig. 2A**). Specificity of the EAAT2 antibody binding was assessed by 5 hours incubation of the undiluted antibody with the immunogenic synthetic peptide corresponding to amino acids 16–31 of rat EAAT2 (Merck Chemicals, Darmstadt, Germany) previous to western blotting (**Fig. 2B**).

### Preparation of rat primary mixed glia cell cultures

Primary cultures of mixed glial cells were prepared from neonatal Sprague–Dawley rat cerebra as described [49], [50]. Briefly, the brains were freed from meninges, and dissociated mechanically and enzymatically with trypsin and DNAse (Sigma-Aldrich, München, Germany). For mixed glial cell cultures, 1×10^5^ cells/well of the single cell suspension were plated directly on poly-L-lysine-coated (Sigma, Deisenhofen, Germany) glass coverslips in 6-well plates. Cells were cultured in standard-medium consisting of DMEM supplemented with 10% fetal calf serum (FCS), 2 mM glutamine, 50 U/ml penicillin, and 50 µg/ml streptomycin (all Biochrom, Berlin, Germany). After 7 days, a confluent astrocytic monolayer developed with both microglia and oligodendrocyte-precursor-cells on top.

### Radioactive glutamate uptake in rat primary mixed glia cell cultures

Uptake of radioactive L-glutamate was measured in the rat primary mixed glial cell culture after 5 days of incubation in standard-media containing various ceftriaxone concentrations (**Fig. 3**). Subsequent determination of uptake was performed using solutions composed of (in mM) 140 NaCl, 4 KCl, 2 CaCl_2_, 1 MgCl_2_, 5 HEPES, pH 7.4 supplemented with 60 µM L-glutamate and 6 nM L-[^3^H]glutamate (Amersham, Freiburg, Germany) according to the known K_m_ value of EAAT2 for glutamate [19] as well as ceftriaxone at the respective concentration. To determine the fraction of total glial uptake mediated by EAAT2, the EAAT2-specific uptake inhibitor dihydrokainate (1 mM; Sigma-Aldrich, München, Germany) was added. To determine the sodium-dependence of the uptake, sodium-free solutions were prepared by substituting sodium with N-methyl-D-glucamine (NMDG). L-[^3^H]glutamate uptake was terminated after 5 min of incubation by 3 times washing with glutamate-free solution, cells were suspended in 0.5% SDS and subjected to scintillation counting after lysis. Uptake of L-[^3^H]glutamate was linear for at least 10 min (data not shown) and pre-incubation with ceftriaxone did not change the average number of cells per well (data not shown).

### Whole cell patch-clamp

Standard whole cell patch-clamp recordings were performed using an Axopatch 200B (Axon Instruments, Union City, CA) amplifier. Borosilicate pipettes were pulled with resistances of 1.0–2.0 megohms. More than 80% of the series resistance was compensated by an analog procedure resulting in calculated voltage errors <5 mV. Currents were filtered at 5 kHz and digitized with a sampling rate of 50 kHz using a Digidata (Axon Instruments) AD/DA converter.

Transient transfection of tsA201 cells with pRCCMV hEAAT2 using the Ca_3_(PO_4_)_2_ technique was performed as previously described [51]. To identify cells with a high probability of expressing recombinant transporters, cells were co-transfected with a plasmid encoding the CD8 antigen, incubated 5 min before use with polystyrene microbeads precoated with anti-CD8 antibodies (Dynabeads M-450 CD 8, Dynal, Great Neck, NY, USA) and detected through a Nikon Eclipse TS100 microscope. The CD8/hEAAT2 cDNA ratio was adjusted to ensure that almost every cell with beads exhibited currents with characteristic properties (**Fig. 4A**).

Cells were clamped to 0 mV for at least 2 s between 10 ms test sweeps to potentials between −200 mV and 100 mV were applied. Current amplitudes were determined 2 ms after the c-peak. The composition of the standard solutions was as follows: extracellular (in mM) 140 NaCl, , 4 KCl, 2 CaCl_2_, 1 MgCl_2_, 5 HEPES, pH 7.4; intracellular (in mM) 115 KCl, 2 MgCl_2_, 5 EGTA, 10 HEPES, pH 7.4. Substrate-containing external solutions were made by adding 500 µM L-glutamate. Extracellular recording solutions contained either 1 mM or 0 mM ceftriaxone and previous to electrophysiological assessment, tsA201 cell transfected with hEAAT2 were incubated over-night in standard-medium containing either 1 mM or 0 mM ceftriaxone as indicated (**Fig. 4B, C**).

### Isolation of splenocytes

Spleens of mice were removed and single cell suspensions were generated by mashing spleens through a 70 µm strainer and lysing red blood cells with ACK buffer. Splenocytes were cultured in DMEM (BioWhittaker, Verviers, Belgium) supplemented with 5% FCS (PAA Laboratories, Pasching, Germany), 10 mM HEPES (Gibco, Invitrogen, Germany), 2 mM L-Glutamine (PAA Laboratories, Pasching, Germany), 50 µM 2-Mercaptoethanol (Gibco, Invitrogen, Germany), 1% Non Essential Amino Acids (BioWhittaker, Verviers, Belgium) and 25 µg/ml Gentamicin (Gibco, Invitrogen, Germany). To obtain certain T cell subsets from isolated splenocytes, T cells were isolated with the respective MACS T cell isolation kit (Miltenyi, Bergisch Gladbach, Germany) according to the manufacturer's manual as indicated.

### Assessment of T cell invasion into the CNS in vivo

Splenocytes of 2D2 TCR-transgenic mice [25] were plated at a densitiy of 1×10^7^/well on a 12 well plate and primed by incubation with MOG_35–55_ (20 µg/ml) on day 1 and 5 and IL-2 (500 IU/ml) on day 1 and 4. On day 3, supernatant medium was taken, analysed for INFγ release using mouse-IFNγ-ELISA according to the manufacturer's protocol (Duoset, R&D Systems, Wiesbaden, Germany) to control for appropriate cytokine release (data not shown) and substituted by fresh medium.

For T cell proliferation analysis, [methyl-^3^H]-thymidine (1.0 µCi/well; Amersham Biosciences, Buckinghamshire, UK) was added to stimulated splenocytes transferred to 96-well plates at a density of 5×10^5^ cells/well for 24 h on day 3 of *in vitro* culture. Afterwards, cells were harvested and [methyl-^3^H]-thymidine incorporation was measured using a liquid scintillation counter (Packard BioScience, Meriden, USA) to control for appropriate proliferation (data not shown).

After 5 days of *in vitro* culture 2D2 splenocytes were collected, washed three times with PBS and 3×10^6^ cells/mice were adoptively transferred via intravenous injection into recipient C57BL/6 mice pre-treated for 5 days with and without ceftriaxone (200 mg/kg i.p.). 4 days after the adoptive transfer, during which no ceftriaxone treatment was performed, brains of recipient mice were removed and the fraction of CNS-invasive cells was analysed via flow cytometry (**Fig. 6**) of 3 to 4 pooled brains per experimental group. Absolute numbers of CD4^+^ T cell per brain were calculated from the absolute number of isolated leukocytes per brain and the fraction of CD4^+^ T cells acquired during FACS analysis.

### Immunophenotyping

For flow cytometric analysis (**Fig. 7A, B**) of lymphocytes isolated from spleen of treated and untreated immunized mice at the disease maximum, single cell suspensions were prepared as described above. Flow cytometry was performed using standard methods. For analysis of T cell subtype distribution cells were stained for 30 minutes with appropriate antibodies or isotype controls (all by BD Bioscience): rat anti-mouse CD4-PerCP (No. 553052), rat anti-mouse CD8a-PE (No. 553033), rat anti-mouse CD44-FITC (No. 553133), rat anti-mouse CD62L-APC (No. 553152). Antigen-presenting cells (APCs) were analysed using the following antibodies (all by BD Bioscience): rat anti-mouse CD11b-PerCP (No. 350993), hamster anti-mouse CD11c-APC (No. 550261), mouse anti-mouse MCHII-FITC (Serotec, No. MCA1501F), rat anti-mouse CD86-FITC (No. 553691), hamster anti-mouse CD80-FITC (No. 553768), hamster anti-mouse CD40-FITC (No. 553723). All antibodies were titrated for optimal staining. Flow cytometry analysis was performed using a FACSCalibur® system (BD Biosciences, Heidelberg, Germany) and results were analyzed using CellQuest Pro (BD Bioscience, Heidelberg, Germany) and FlowJo (Treestar, USA) software.

### Generation of murine dendritic cells

Dendritic cells (DCs) from WT C57BL/6 mice were prepared as described [52], [53]. In brief, bone marrow cells were flushed out of femur and tibia bones with PBS. Cells were incubated for 30 s with ACK buffer (0.15 M NH_4_Cl, 10 mM KHC0_3_, 0.1 mM EDTA) and filtered through a 70-µm cell strainer. The single-cell suspension was cultured in RPMI 1640 medium (BioWhittaker, Verviers, Belgium) supplemented with 10% FBS, 2 mM L-glutamine (PAA Laboratories, Pasching, Germany), 50 µM 2-mercaptoethanol, antibiotics (100 U/ml penicillin/10 µg/mL streptomycin; Biochrom, Berlin, Germany) and 20 ng/ml mGM-CSF (Peprotech, Hamburg, Germany). On days 3 and 6 medium containing 20 ng/ml mGM-CSF or 10 ng/ml mGM-CSF was added. Bone marrow-derived dendritic cells were harvested on day 8 and characterized as more than 85% pure CD11c^+^ cells. Cells were frozen, kept in liquid nitrogen and thawn on demand.

### T cell activation and antigen-recall assays

For antigen-recall experiments (**Fig. 7C, D**), 1×10^6^ splenocytes/well from permanently and therapeutically ceftriaxone-treated and untreated MOG-immunized mice were cultured on 24-well plates with 10 µg/ml MOG_35–55_-peptide for 72 h. As control, splenocytes from the same mice were activated with anti-CD3/CD28 beads (Dynal Biotech, Oslo, Norway) at a ratio of 1∶1 or left untreated. After 72 h, IFNγ-levels were determined in the supernatants by mouse-IFNγ-ELISA according to the manufacturer's protocol (Duoset, R&D Systems, Wiesbaden, Germany).

For T cell proliferation analysis (**Fig. 8A**), isolated CD4^+^ T cells from the spleen of non-immunized mice were stimulated with anti-CD3/CD28 beads (Dynal Biotech, Oslo, Norway) for 72 h, respectively, and 24 h before the end of incubation, [methyl-3H]-thymidine (1.0 µCi/well; Amersham Biosciences, Buckinghamshire, UK) was added. Cells were harvested and [methyl-3H]-thymidine incorporation was measured using a liquid scintillation counter (Packard BioScience, Meriden, USA).

To asses the effect of ceftriaxone on antigen-presentation separated from T cell activation and proliferation (**Fig. 8B, C**), dendritic cells (DCs; 2×10^6^/well on 24 well plates) isolated from non-immunized mice were pulsed for 12 h with of MOG_35–55_ peptide (50 µg/ml) in the absence and presence of 100 and 500 µM ceftriaxone. Subsequently, DCs were washed and 1×10^5^ DCs per well were co-cultured for 3 days with 5×10^5^ syngeneic MOG-reactive CD4^+^ T cells obtained from MOG-immunized mice at the disease maxium on 48-well plates (ratio of TC/DC = 5∶1). The supernatants were analyzed for IL2, IFNγ, IL4, and IL17 production by ELISA according to the manufacturer's protocol.

All experiments were performed as triplicates.

### Statistical analysis

All results are presented as mean±SEM. Statistical analysis was performed using the student's t-test modified for small samples [54]. P-values ≤0.05 were considered significant (*). P-values ≤0.01 and ≤0.001 were considered highly significant (** and ***, respectively).
