# Correction: A β-Lactam Antibiotic Dampens Excitotoxic Inflammatory CNS Damage in a Mouse Model of Multiple Sclerosis

**DOI:** 10.1371/annotation/b898d6ee-801a-474e-9776-72e4da664b89

**Published:** 2008-09-25

**Authors:** Nico Melzer, Sven G. Meuth, Delany Torres-Salazar, Stefan Bittner, Alla L. Zozulya, Christian Weidenfeller, Alexandra Kotsiari, Martin Stangel, Christoph Fahlke, Heinz Wiendl

There was a factual error in the first two sentences of the second paragraph of the Discussion section. The correct text should read: Moreover, ceftriaxone had no direct effect on T cell proliferation, but had a tremendous effect on T cell migration into the CNS and indirectly hampered their proliferation and proinflammatory IFNγ and IL17 secretion through modulation of myelin-antigen presentation by antigen-presenting cells.

