# Correction: A β-Lactam Antibiotic Dampens Excitotoxic Inflammatory CNS Damage in a Mouse Model of Multiple Sclerosis

**DOI:** 10.1371/annotation/b1ff1c14-fa84-40a0-b095-e5ee47c74125

**Published:** 2011-01-10

**Authors:** Nico Melzer, Sven G. Meuth, Delany Torres-Salazar, Stefan Bittner, Alla L. Zozulya, Christian Weidenfeller, Alexandra Kotsiari, Martin Stangel, Christoph Fahlke, Heinz Wiendl

The authors would like to add the following to the Competing Interests section: "Dr. Wiendl has received funding for travel and speaker honoraria from Bayer Schering Pharma, Biogen Idec/Elan Corporation, Sanofi-Aventis, Merck Serono, and Teva Pharmaceutical Industries Ltd.; has served/serves as a consultant for Merck Serono, Medac, Inc., Sanofi-Aventis/Teva Pharmaceutical Industries Ltd., Biogen Idec, Bayer Schering Pharma, Novartis, and Novo Nordisk; and receives research support from Bayer Schering Pharma, Biogen Idec/Elan Corporation, Sanofi-Aventis, Merck Serono, and Novo Nordisk."

